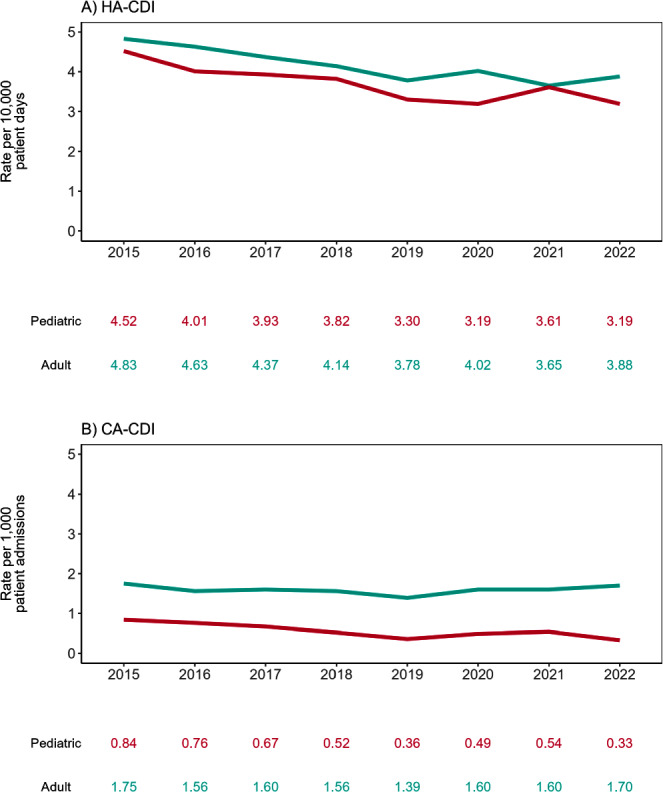# Molecular and Epidemiological Characterization of Pediatric and Adult C. difficile Infection in Canadian Hospitals, 2015-2022

**DOI:** 10.1017/ash.2024.108

**Published:** 2024-09-16

**Authors:** Timothy Du, Kelly Baekyung Choi, Anada Silva, Cassandra Lybeck, George Golding, Romeo Hizon, Sean Ahmed, Blanda Chow, Ian Davis, Meghan Engbretson, Gerald Evans, Charles Frenette, Jennie Johnstone, Pamela Kibsey, Kevin Katz, Joanne Langley, Jenine Leal, Bonita Lee, Yves Longtin, Dominik Mertz, Jessica Minion, Michelle Science, Jocelyn Srigley, Kathryn Suh, Reena Titoria, Nisha Thampi, Alice Wong, Jeannette Comeau, Susy Hota

**Affiliations:** Public Heath Agency of Canada; Infection Prevention & Control, Alberta Health Services; Queen Elizabeth II Health Sciences Centre; Children’s Hospital of Eastern Ontario; Kingston Health Sciences Centre; McGill University Health Center; Island Health Vancouver Island; North York General Hospital; Dalhousie University; Alberta Health Services/University of Calgary; University of Alberta; Jewish General Hospital; McMaster University; Regina Qu'Appelle Health Region, Regina, SK; The Hospital for Sick Children; BC Children’s & BC Women’s Hospitals; The Ottawa Hospital; PHSA; Children’s Hospital of Eastern Ontario; Royal University Hospital; University Health Network

## Abstract

**Background:** The molecular and epidemiological landscape of C. difficile infection (CDI) has evolved markedly in the last decade; however, limited information is available contrasting differences between adult and pediatric populations. We describe a multicenter study evaluating healthcare-associated (HA) and community-associated (CA) adult and pediatric-CDI identified in the Canadian Nosocomial Infection Surveillance Program (CNISP) network from 2015 to 2022. **Methods:** Hospitalized patients with CDI were identified from up to 84 hospitals between 2015–2022 using standardized case definitions. Cases were confirmed by PCR, cultured, and further characterized using ribotyping and E-test. We used two-tailed tests for significance (p≤0.05). **Results:** Of 30,817 cases reported, 29,245 were adult cases [HA-CDI (73.2%), CA-CDI (26.8%)] and 1,572 were pediatric cases [HA-CDI (77.7%), CA-CDI (22.3%)]. From 2015 to 2022, HA-CDI rates decreased 19.7% (p=0.007) and 29.4% (p=0.004) in adult and pediatric populations, respectively (Figure 1). CA-CDI rates remained relatively stable in the adult population (p=0.797), while decreasing 60.7% in the pediatric population (p=0.013). Median ages of adult and pediatric patients were 70 (interquartile range (IQR), 58–80) and seven (IQR, 3–13) years, respectively. Thirty-day all-cause mortality was significantly higher among adult vs. Pediatric CDI patients (11.0% vs 1.4%, p < 0.0001). No significant differences in other severe outcomes were found. Ribotyping and susceptibility data were available for 4,620 samples: 3,558 adult (77.0%) and 1,062 pediatric (23.0%). The predominant adult and pediatric ribotypes (RT) were 106 (12.2/16.2%), 027 (11.4/3.2%), and 014 (8.8/8.2%). Overall, RT027 prevalence significantly decreased from 17.9% in 2015 to 3.2% in 2022 (p=0.003), while RT106 increased from 8.5% to 14.4%. Resistance rates among adult and pediatric isolates were similar for all antimicrobials tested except moxifloxacin (16.2% vs. 6.2%, p < 0.0001, respectively). Adult moxifloxacin resistance decreased from 30% to 6.3% from 2015 to 2022 (p=0.006). Adults with moxifloxacin-resistant CDI were older (median: 74 vs. 69 years, p < 0.001) and had higher thirty-day all-cause mortality (13% vs. 9.8%, p=0.041) and recurrence (10% vs. 5.7%, p < 0.001) compared to those with moxifloxacin non-resistant CDI, while these trends were not observed in pediatric patients. Among RT027 strains, moxifloxacin resistance decreased from 91.0% in 2015 to 7.1% in 2022. There was one metronidazole-resistant pediatric sample in 2018 and no resistance to vancomycin or tigecycline in either population. **Conclusion:** We have found differences in the epidemiological and molecular characteristics of adult and pediatric CDI, with higher thirty-day all-cause mortality among adults. Overall, RT106 has replaced RT027 as the predominant ribotype with a concomitant decrease in fluoroquinolone resistance.

**Figure:**